# Supplementary material for: In Vivo Evaluation of (−)-Zampanolide Demonstrates Potent and Persistent Antitumor Efficacy When Targeted to the Tumor Site
Source: Molecules. 2022 Jul 1;27(13):4244. doi: 10.3390/molecules27134244 (PMC9268097; doi:10.3390/molecules27134244)

In vivo evaluation of (-)-zampanolide demonstrates potent and persistent antitumor efficacy when targeted to the tumor site

Leila Takahashi-Ruiz,[†] Joseph D. Morris,[‡] Phillip Crews,[§] Tyler A. Johnson,[‡,*] and April L. Risinger,[†*]

[†]*Department of Pharmacology, University of Texas Health Science Center at San Antonio, Texas 78229, USA*

[‡]*Department of Natural Sciences & Mathematics, Dominican University of California, California 94901, USA*

[§]*Department of Chemistry & Biochemistry, University of California, Santa Cruz, California 95064, USA*

Supporting Information

[Contents]

Figure S1. ^{1}H NMR spectrum of (-)-zampanolide in benzene-d6 at 400 MHz page S2
Figure S2. ^{13}C NMR spectrum of (-)-zampanolide in benzene-d6 at 100 MHz page S3

Figure S1. ^{1}H NMR spectrum of (-)-zampanolide in benzene-d6 at 400 MHz

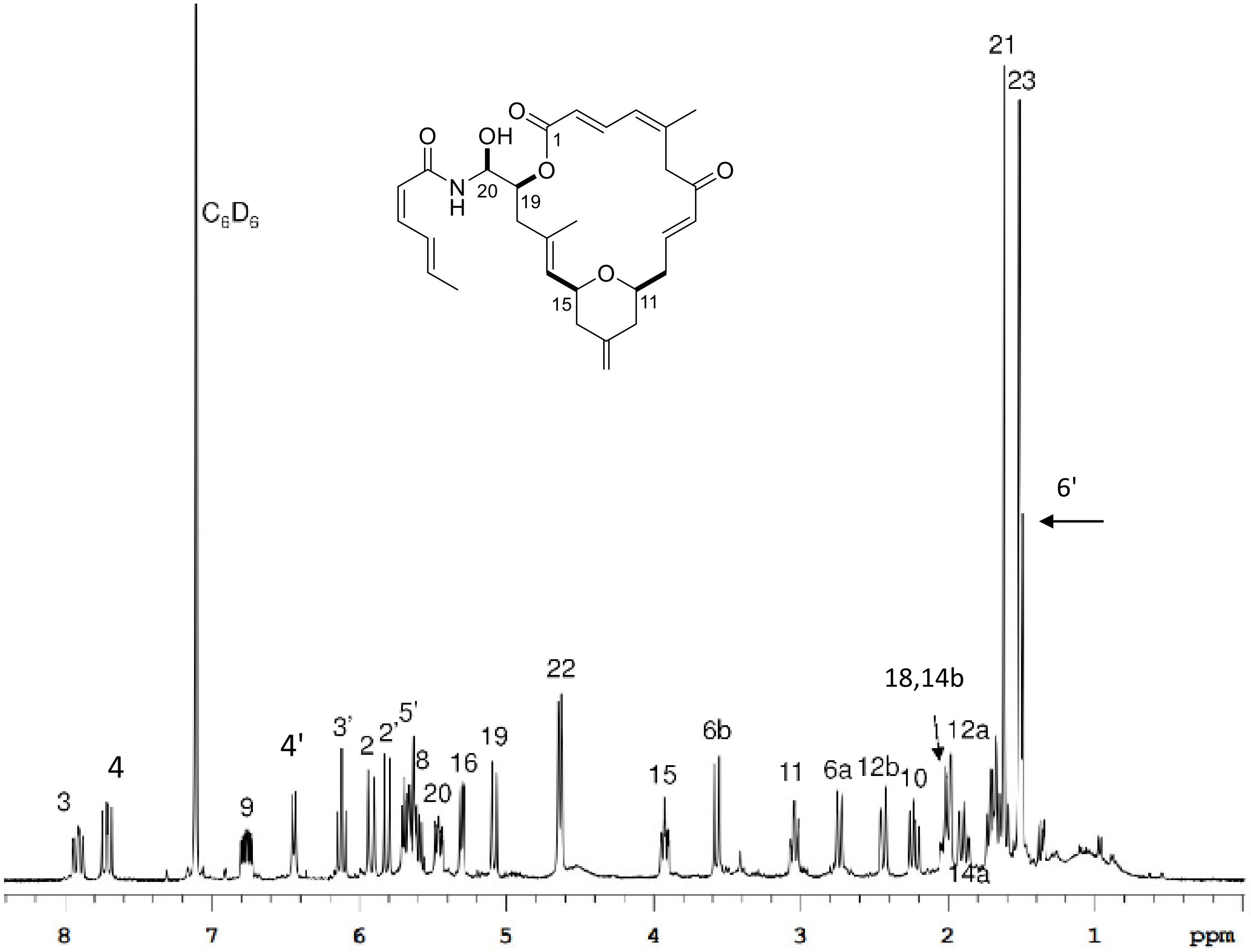

Figure S2. ^{13}C NMR spectrum of (-)-zampanolide in benzene-d6 at 100 MHz

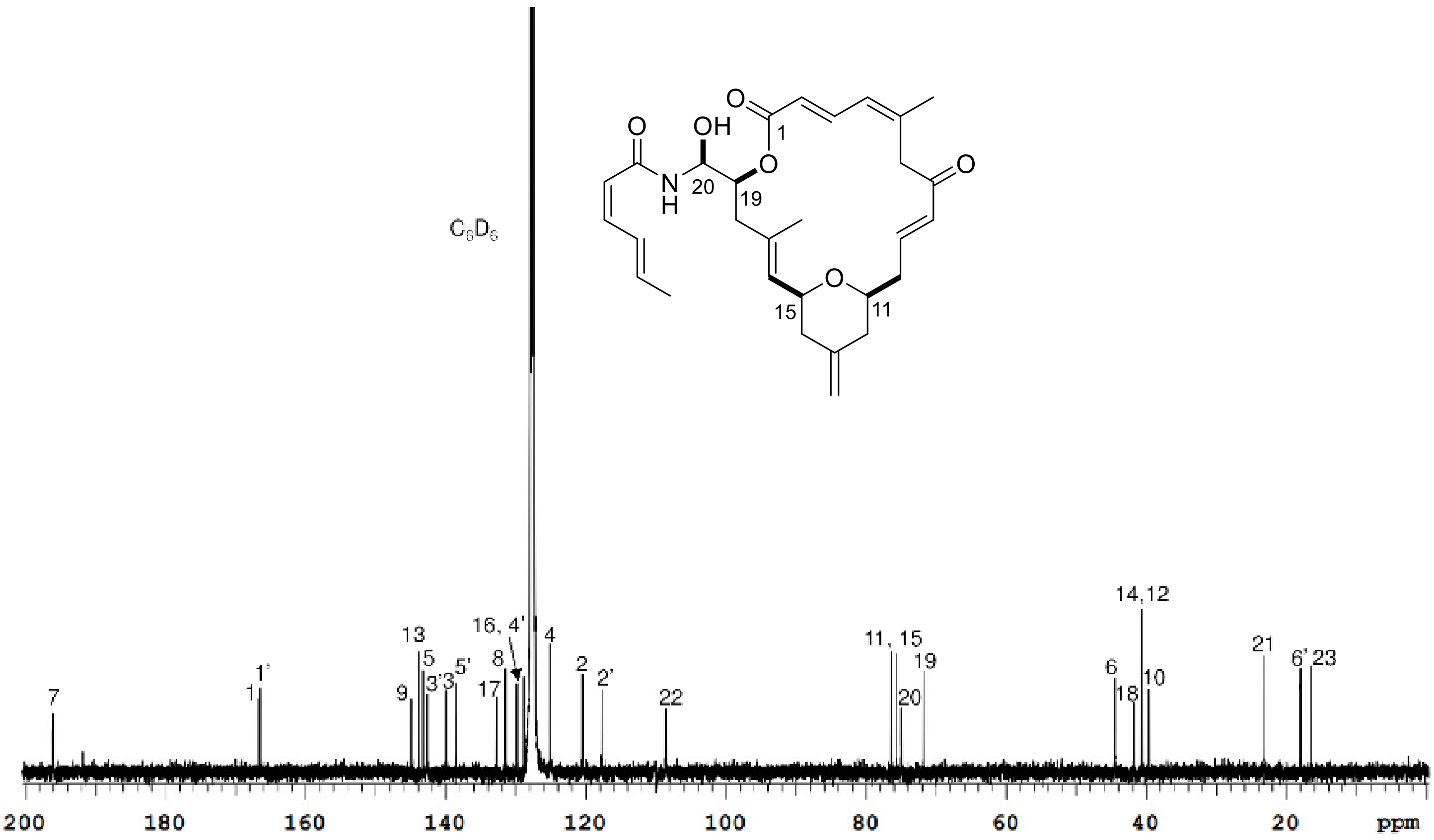

Supplement: Supplementary file 1 [file molecules-27-04244-s001.zip › Zampanolide Manuscript Supplemental Figures FINAL 5.16.22.pdf]
